# Phage-directed precision microbiome medicine: engineering pathobiont depletion, synbiotic succession, microbial-function editing, metabolite host signaling, neurophagomics, and durable ecological state control in gut dysbiosis associated diseases

**DOI:** 10.3389/fmicb.2026.1964616

**Published:** 2026-10-22

**Authors:** Umar Saeed, Zahra Zahid Piracha

**Affiliations:** 1 Korea University College of Health Sciences, Korea University, Seoul, Republic of Korea; 2 Institute of Graduate Studies and Research, Cyprus International University, Nicosia, Cyprus; 3 Széchenyi István University, Győr, Hungary; 4 Center for Research Development, University of Cyberjaya, Persiaran Bestari, Cyberjaya, Selangor, Malaysia

**Keywords:** bacteriophage, CRISPR, dysbiosis, ecological succession, Eco-PK/PD, gut microbiome, pathobiont, phageome

## Abstract

Bacteriophages are being investigated as strain-selective tools for modifying intestinal microbial communities, but the evidence supporting ecological reconstruction is substantially less mature than the evidence supporting targeted bacterial depletion. This review separates current knowledge into three domains: established and emerging experimental evidence, human translational evidence, and hypotheses proposed for future testing. Human studies show that orally administered natural or engineered phages can be recovered from the gastrointestinal tract and can alter selected bacterial populations without antibiotic-like loss of overall community diversity. Preclinical studies further demonstrate targeted suppression of inflammatory *Klebsiella pneumoniae* in inflammatory bowel disease models, cytolytic *Enterococcus faecalis* in alcohol-associated liver disease, evolutionary steering of *Escherichia coli*, in situ bacterial genome editing, local therapeutic-protein production, and phage-probiotic cooperation. These findings do not yet establish that an integrated phage-successor-prebiotic sequence can durably reprogram a human gut ecosystem. To organize this translational gap, we use Subtract-Seed-Feed-Lock as an explicitly proposed design framework. A phage or phage-derived platform first depletes, weakens, or edits a disease-relevant bacterial strain or function; a niche-matched successor is then introduced; a selectively utilized nutrient supports that successor; and durability is assessed after treatment withdrawal. Related terms used in this review—Functional Phageability, Minimum Ecological Dose, Ecological Vacancy Window, nutrient-conditioned phagogram, ecological pharmacokinetics/pharmacodynamics (Eco-PK/PD), ecological pharmacovigilance, neurophagomic reverse translation, and microbiome-state digital twin—are hypothesis-generating operational concepts rather than validated clinical methods. Host signaling is likewise framed as predominantly indirect: phage-induced changes in bacterial abundance or function may alter lipopolysaccharide, short-chain fatty acids, indoles, bile acids, and other metabolites that subsequently engage inflammatory, barrier, hepatic, or neural pathways. The gut-brain application remains especially preliminary; targeting *urdA*-positive imidazole-propionate producers has not been validated with therapeutic phages. Clinical development will require reproducible good manufacturing practice, control of endotoxin and host-cell contaminants, product stability, resistance and immune-neutralization monitoring, environmental-shedding assessment, and regulatory strategies for genetically modified, dynamically updated, and multi-component products. The immediate opportunity is therefore precise microbial perturbation with evidence-matched endpoints; durable programmed reconstruction remains an experimentally testable objective.

## 1 Introduction

### 1.1 Scope, terminology, and evidence architecture

The modern revival of phage therapy has been driven largely by antimicrobial resistance, for which the central objective is often clearance of an infecting pathogen. Gut dysbiosis presents a different problem. A disease-associated strain may be a long-term resident embedded within nutrient exchange, phage-prophage interactions, spatial niches, mucosal immunity, and host metabolism. Selective removal can therefore create an ecological opportunity without necessarily restoring a beneficial community. Experimental phage perturbation can also alter non-target taxa and the metabolome, and synthetic communities can occupy alternative stable states (Hsu et al., 2019; Rios Garza et al., 2026). These observations justify an ecological perspective, but they do not by themselves demonstrate controllable state switching in patients.

This review has three deliberately separated functions. First, it synthesizes established and emerging experimental evidence on gut phage biology and engineered phage platforms. Second, it evaluates human and translational evidence, including oral phage trials, disease-targeting studies, delivery, safety, and manufacturing. Third, it presents a limited set of author-proposed operational frameworks that can be falsified experimentally. The central proposal is Subtract-Seed-Feed-Lock; the other proposed terms are subordinate tools for selecting combinations, measuring their behavior, and testing durability (Figure 1). None should be interpreted as an established clinical methodology.

**Figure 1:**
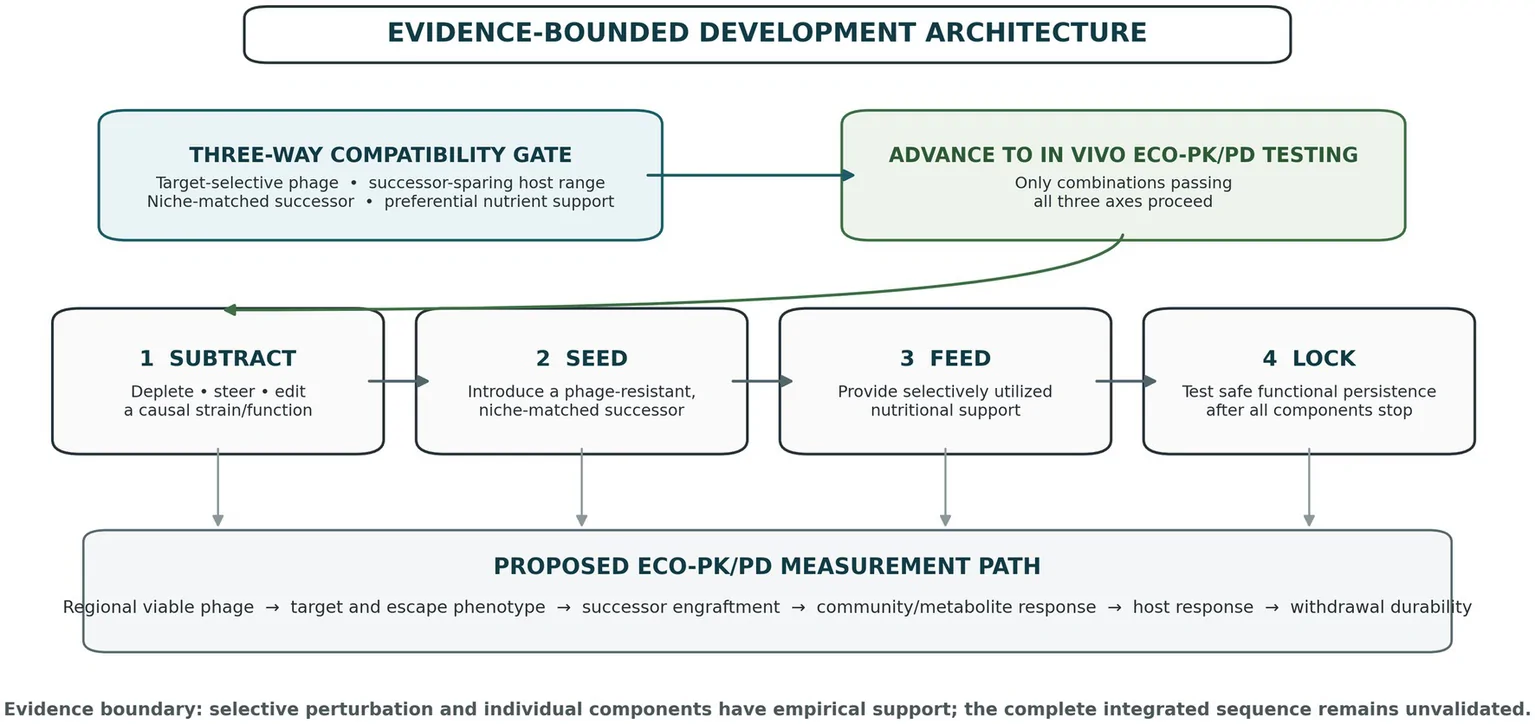
Evidence-bounded framework for precision phage-directed gut microbiome engineering. Established and emerging evidence supports several forms of selective phage perturbation, whereas the complete Subtract-Seed-Feed-Lock sequence is an author-proposed hypothesis. Successor and prebiotic selection must pass a three-way compatibility gate before *in vivo* Eco-PK/PD evaluation. Durability is assessed after withdrawal of all active components. Conceptual synthesis based on phage perturbation, alternative-state, engraftment, and successor-competition studies (Hsu et al., 2019; Zmora et al., 2018; Bakkeren et al., 2025; Lentsch et al., 2025; Rios Garza et al., 2026; Goswami et al., 2026).

In this review, virome denotes the entire viral community detected in a sample, including bacteriophages and eukaryotic viruses. Phageome is used only for the bacteriophage component. Prophage denotes a phage genome integrated into, or stably maintained within, a bacterial host. Because study designs differ markedly, claims are labeled according to a five-tier evidence classification (Table 1). The tier indicates study type and translational proximity, not a formal certainty-of-evidence score.

**Table 1:** Evidence classification applied throughout this review.

Tier	Evidence class	Typical study design	Permitted interpretation in this review
1	Human interventional evidence	Randomized trial, controlled clinical study, or first-in-human intervention	Demonstrates human feasibility, safety, pharmacology, or efficacy only for the tested product, population, and endpoint
2	Human observational evidence	Cohort, case–control, cross-sectional multi-omics, or longitudinal virome study	Supports association, stratification, or target nomination; does not establish therapeutic causality
3	Preclinical therapeutic evidence	Mouse, chick, zebrafish, organoid, *ex vivo* community, or other intervention model	Supports biological proof of concept; species and model limitations must be stated
4	Mechanistic evidence	Cell culture, biochemical, molecular, genetic, or receptor-pathway study	Supports mechanism or plausibility; does not establish clinical benefit

## 2 Established and emerging experimental evidence

### 2.1 Selective perturbation within a coupled bacterial-viral ecosystem

Gut dysbiosis cannot be reduced to a fixed list of beneficial and harmful species. Disease-relevant properties may be strain restricted; bacterial receptors change with oxygen tension, mucus, bile acids, and nutrients; and narrow phage host range can be advantageous or limiting. In a mouse model (Tier 3), selective phage perturbation altered both non-target bacterial populations and metabolites (Hsu et al., 2019). This community response makes the ecological trajectory—not only the fall in the targeted colony-forming units—a relevant outcome.

The endogenous phageome is also dynamic. Long-read human metagenomics (Tier 2) indicates that many prophage-host relationships are longitudinally stable while a minority is gained or lost within persistent bacterial hosts (Wirbel et al., 2026). Culture and synthetic-community experiments (Tier 3) show that host and community context changes prophage inducibility (Dahlman et al., 2025). Inflammatory compounds can induce commensal prophages, and experimental colitis is accompanied by altered temperate-phage dynamics (Sinha et al., 2026). Communication systems can further coordinate or interfere with lysis-lysogeny decisions across phages (Manley et al., 2026; Gallego-del-Sol et al., 2026). Therapeutic perturbation may therefore alter prophage induction, bacterial competition, and horizontal gene transfer even when the administered product is strictly lytic.

Lysogeny is not intrinsically detrimental. A human gut prophage landscape identified a prophage-associated *futC* fucosyltransferase pathway linked to 2′-fucosyllactose production and protection in an experimental colitis model (Tier 3) (Gao et al., 2025). The relevant safety question is consequently functional: which prophages mobilize harmful cargo, which confer beneficial functions, and how does treatment change their state?

### 2.2 Modes of phage-directed intervention

Natural lytic phages remain the most direct means of depletion, but emerging platforms can change the therapeutic unit from a species to a strain, gene, receptor, or secreted function. Receptor-binding-protein engineering can retarget adsorption. CRISPR-Cas payloads can provide genotype-selective killing. Non-replicating phage-derived particles can deliver base editors, and CRISPR-associated transposases can inactivate virulence while installing a payload. Other platforms use resident bacteria as transient local bioreactors, apply phage-derived lysins for non-replicating pruning, or select escape mutations that reduce virulence or fitness. These modalities and their evidence levels are summarized together (Table 2; Figure 2).

**Table 2:** Experimental and translational phage platforms for gut microbiome manipulation.

Platform	Primary mechanism	Intended ecological effect	Representative evidence	Evidence tier/maturity
Natural lytic phage	Receptor-specific infection and lysis	Selective strain depletion	IBD-associated *K. pneumoniae* and cytolytic *E. faecalis* targeting (Federici et al., 2022; Duan et al., 2019)	Tier 3; therapeutic proof of concept in mice
RBP/tail-fiber engineered phage	Retargeted adsorption	Narrowed or redirected host range	SNIPR001 development (Petersen et al., 2026; Gencay et al., 2024)	Tier 1 safety/pharmacology plus Tier 3 efficacy
CRISPR-Cas phage	Sequence-specific bacterial killing	Genotype-selective subtraction	SNIPR001 (Petersen et al., 2026; Gencay et al., 2024)	First-in-human Phase I; efficacy not established
Phage-derived base editor	Non-replicating genetic payload	Function editing without bacterial killing	In-gut *E. coli* editing (Brödel et al., 2024)	Tier 3 mouse proof of concept
CRISPR-associated transposase	Virulence-gene inactivation and payload insertion	Phenotypic conversion	Enterobacteriaceae platform (Ronda et al., 2025)	Tier 3 preclinical
Therapeutic-protein phage	Infection-dependent local protein production	Local biologic delivery	Engineered T4-serpin system (Baker et al., 2026)	Tier 3 mouse proof of concept
Endolysin	Enzymatic cell-wall cleavage	Temporary, non-replicating ecological pruning	Selective *Streptococcus salivarius* reduction (Garneau et al., 2026)	Tier 3 *ex vivo*/experimental
Evolutionary-steering phage	Escape through costly receptor alteration	Reduced virulence or competitiveness	Capsule-loss steering in *E. coli* K1 (Larsson et al., 2026)	Tier 3 mouse proof of principle
Mucosa-retentive phage	Ig-like adhesin-mediated epithelial association	Increased regional residence	Transferable phage adhesin (Apjok et al., 2026)	Tier 3 mouse/cell evidence
Defined multi-phage program	Coordinated activity of characterized phages	Community-level perturbation	Enabled by cultured gut phage resources (Xing et al., 2025)	Platform-enabling; not a validated therapeutic class

**Figure 2:**
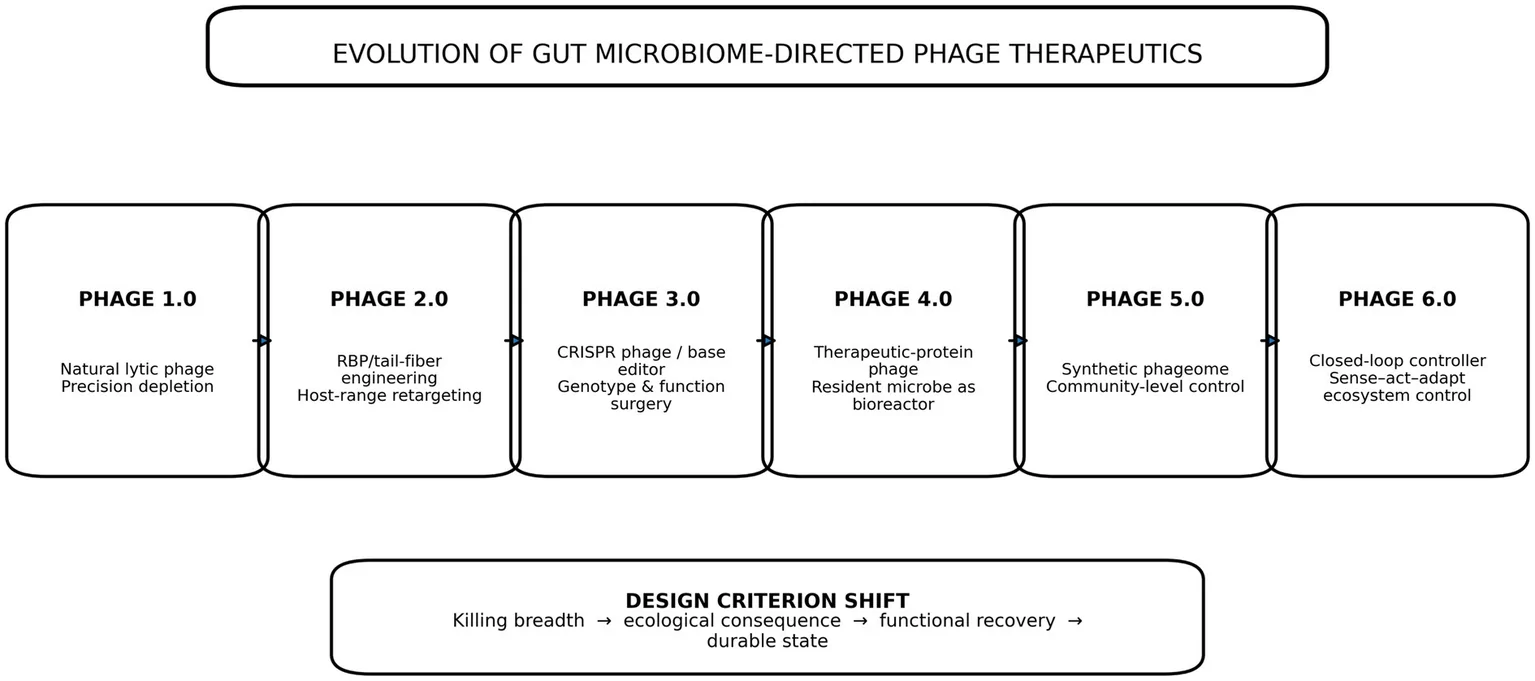
Evolution of gut microbiome-directed phage platforms. The diagram distinguishes demonstrated modalities from the proposed progression toward closed-loop ecological control. Natural lysis, engineered host range, CRISPR killing, *in situ* editing, and local protein delivery have experimental or early human support; community-level state control remains hypothetical (Federici et al., 2022; Petersen et al., 2026; Gencay et al., 2024; Brödel et al., 2024; Ronda et al., 2025; Baker et al., 2026; Xing et al., 2025; Larsson et al., 2026; Garneau et al., 2026).

## 3 Human translational evidence

Human evidence remains limited and is concentrated in safety, tolerability, gastrointestinal recovery, and selected microbiome outcomes rather than disease-modifying ecological reconstruction. The PHAGE study tested the four-phage PreforPro preparation (LH01, LL5, T4D, and LL12) in adults with mild gastrointestinal symptoms and reported selected bacterial changes without broad loss of community diversity (Tier 1) (Febvre et al., 2019; Gindin et al., 2019). PHAGE-2 paired PreforPro with *Bifidobacterium animalis* subsp. *lactis* BL04 (Grubb et al., 2020). The PHAGE-2 report, not the PHAGE-3 registry, found no clear evidence that phage co-administration increased BL04 survival, although some gastrointestinal and microbiome outcomes differed between groups. This result does not support a general claim that phages enhance probiotic engraftment.

PHAGE-3 instead paired PreforPro with *Bacillus subtilis* DE111; the registry describes a randomized, triple-masked study, but results were not posted in the source reviewed for this manuscript (ClinicalTrials.gov, 2024). Synbiotic+ combines the same four *E. coli*-targeting phages with *Lacticaseibacillus rhamnosus* GG, *B. animalis* subsp. *lactis* BB-12, and tributyrin (ClinicalTrials.gov, 2026a). These programs show increasing interest in multi-component formulations but do not yet establish clinical superiority of integrated phage-probiotic-postbiotic treatment.

SNIPR001 provides a distinct first-in-human precedent. In Phase I, four orally administered engineered CRISPR-Cas phages were recovered from stool in a dose-associated manner, remained predominantly gastrointestinal, and did not cause detectable global microbiome disruption relative to placebo (Tier 1) (Petersen et al., 2026). These results support short-term safety, recovery, and taxon-selective pharmacology; they do not establish efficacy for chronic dysbiosis. An Ib/IIa program is evaluating intestinal *E. coli* reservoir control in allogeneic hematopoietic stem-cell transplantation (ClinicalTrials.gov, 2026b). The scope and limitations of current human programs are summarized together (Table 3; Figure 3).

**Table 3:** Human clinical development relevant to phage-microbiome and phage-probiotic therapy.

Program	Phage intervention	Probiotic/co-intervention	Design and status described in cited source	Evidence-supported interpretation
PHAGE / NCT03269617	LH01 + LL5 + T4D + LL12	None	Randomized crossover; completed	Oral tolerability and selective microbiota modulation; broad diversity largely preserved (Febvre et al., 2019; Gindin et al., 2019)
PHAGE-2 / NCT04511221	PreforPro, 10^6^ PFU/day	*B. animalis* BL04, 10^9^ CFU/day	Randomized, triple-masked	Some functional benefits; no clear increase in probiotic survival (Grubb et al., 2020)
PHAGE-3 / NCT05750433	PreforPro	*B. subtilis* DE111	Randomized, triple-masked; completed	Designed to test combined effects; registry results not posted in the reviewed source (ClinicalTrials.gov, 2024)
Synbiotic+ / NCT06834984	PreforPro, 10^6^ PFU	LGG + BB-12 + tributyrin	Randomized, triple-masked; registered program	Translational example of phage-probiotic-postbiotic integration; efficacy not established (ClinicalTrials.gov, 2026a)
SNIPR001 Phase I	Four engineered CRISPR-Cas phages	None	First-in-human dose escalation	Short-term safety, gastrointestinal recovery, and microbiome-sparing precedent (Petersen et al., 2026)
SNIPR001 Ib/IIa / NCT06938867	SNIPR001	Standard-of-care context	Intestinal *E. coli* reservoir in allogeneic HSCT	Ongoing translation toward selective reservoir control; no efficacy conclusion yet (ClinicalTrials.gov, 2026b)

**Figure 3:**
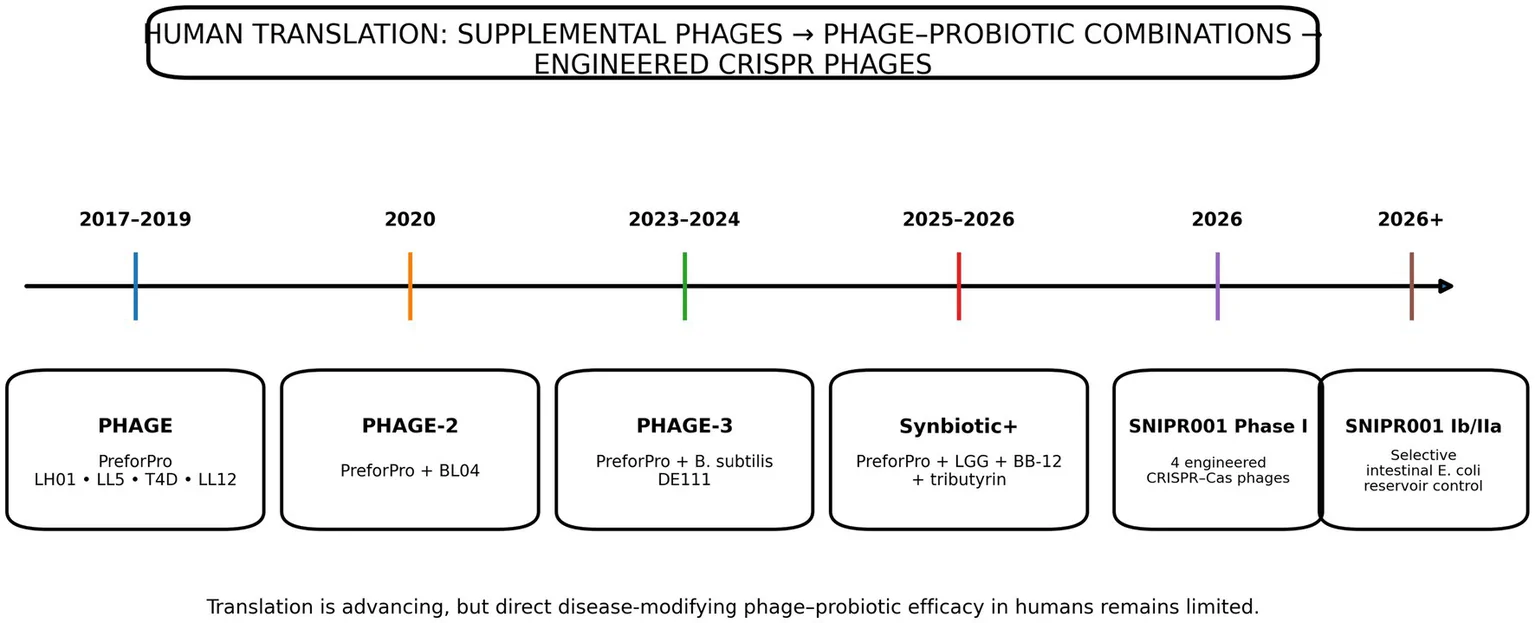
Human translational trajectory from supplemental gut phages to engineered and multi-component products. The timeline denotes clinical development events, not increasing efficacy. Direct disease-modifying phage-probiotic efficacy in humans has not been demonstrated (Febvre et al., 2019; Gindin et al., 2019; Grubb et al., 2020; Petersen et al., 2026; ClinicalTrials.gov, 2024; ClinicalTrials.gov, 2026a; ClinicalTrials.gov, 2026b).

## 4 Host pathways potentially modulated through phage-induced ecological restructuring

### 4.1 An indirect causal chain should organize interpretation

The most defensible mechanistic model is indirect: a phage changes bacterial abundance, genotype, or activity; this modifies microbial structural products or metabolites; those intermediates then engage host receptors and intracellular pathways. Evidence that a microbial molecule regulates Toll-like receptor 4 (TLR4), NOD2, the NLRP3 inflammasome, G-protein-coupled receptors, the aryl hydrocarbon receptor (AhR), pregnane X receptor (PXR), or bile-acid receptors is not equivalent to evidence that a phage intervention normalizes that pathway. The evidence boundaries are summarized graphically and in tabular form (Figure 4; Table 4).

**Figure 4:**
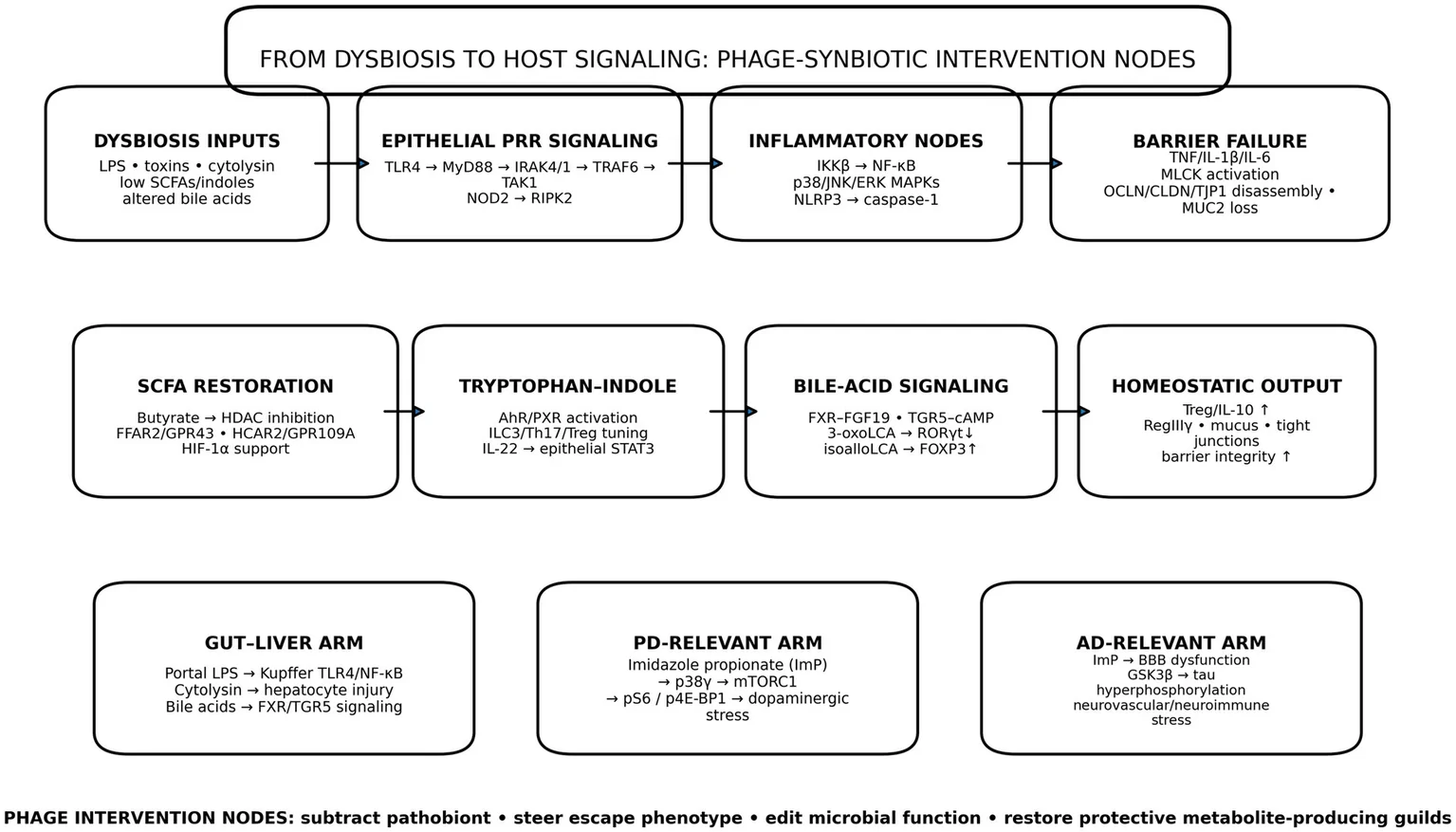
Host pathways potentially affected by phage-induced ecological restructuring. Solid evidence supports microbiota-to-host signaling through inflammatory ligands, short-chain fatty acids, indoles, and bile acids; dashed therapeutic interpretation indicates that phages may influence these pathways indirectly by changing the responsible bacterial populations or functions. Direct pathway normalization by an integrated phage-synbiotic intervention remains to be demonstrated (Furusawa et al., 2013; Singh et al., 2014; Kelly et al., 2015; Zelante et al., 2013; Venkatesh et al., 2014; Hang et al., 2019; Song et al., 2020; Mann et al., 2024; Won et al., 2025; Older et al., 2024; Podlacha et al., 2024; Bichet et al., 2023; Stallhofer et al., 2026; Cooney et al., 2010; Travassos et al., 2010; Adolph et al., 2013; Pols et al., 2011).

**Table 4:** Evidence boundaries for microbiota-host pathways relevant to precision phage therapy.

Microbial or phage input	Host pathway/node	Established biological consequence	Phage-specific evidence	Appropriate interpretation
Phage DNA/capsid	TLR9-MyD88-IRF7/NF-κB; cGAS-STING; AKT/actin	Context-dependent uptake and immune or metabolic responses	Chicken intervention and mammalian-cell studies (Podlacha et al., 2024; Bichet et al., 2023)	Direct interaction is a safety/PK variable; direction cannot be generalized
Muramyl dipeptide/intracellular bacteria	NOD2-RIPK2; ATG16L1; UPR	Xenophagy, Paneth-cell defense, epithelial stress	No precision phage-synbiotic causality	Mechanistic rationale for strain/function selectivity (Cooney et al., 2010; Travassos et al., 2010; Adolph et al., 2013)
Lipopolysaccharide/PAMP excess	TLR4-MyD88-IRAK-TRAF6-TAK1	NF-κB/MAPK activation, inflammatory cytokines, barrier injury	Plausible after Gram-negative depletion; rarely measured as a causal sequence	Candidate indirect readout (Older et al., 2024; Iliev et al., 2025)
Cellular stress/bacterial products	NLRP3-ASC-caspase-1	IL-1β/IL-18 maturation and pyroptotic amplification	No integrated intervention evidence	Proposed downstream inflammatory endpoint
Butyrate and other SCFAs	HDAC; FFAR2/GPR43; HCAR2/GPR109A; HIF	Regulatory T cells and epithelial support	Microbiota biology established; phage-mediated restoration unproven	Measure metabolite flux and pathway response (Furusawa et al., 2013; Singh et al., 2014; Kelly et al., 2015; Mann et al., 2024)
Indole derivatives	AhR; PXR; IL-22-JAK/STAT3	Mucosal repair, mucus, antimicrobial programs	No direct precision-phage demonstration	Preserve or restore indole-producing functions (Zelante et al., 2013; Venkatesh et al., 2014)
Secondary bile acids	FXR; TGR5; RORγt	Enterohepatic signaling and regulatory T-cell/TH17 balance	Human association and mechanistic evidence; phage causality absent	Measure bile-acid composition and receptor activity (Hang et al., 2019; Song et al., 2020; Won et al., 2025; Stallhofer et al., 2026; Pols et al., 2011)
Imidazole propionate	p38γ-mTORC1; endothelial barrier; GSK3β	Parkinsonian and Alzheimer-related phenotypes in experimental systems	Therapeutic phage not tested	Hypothesis-generating target discovery only (Park et al., 2025; Vemuganti et al., 2026)

Direct phage-mammalian signaling is context dependent. In a chicken therapy model (Tier 3), phage double-stranded DNA activated a TLR9-MyD88 axis with IRF7/NF-κB-linked interferon-β responses, while cGAS-STING signaling was initiated but interrupted at IRF3 phosphorylation (Podlacha et al., 2024). In cultured mammalian cells (Tier 4), highly purified T4 phage entered macropinocytic compartments without activating TLR9 or cGAS-STING; phosphoproteomics instead implicated AKT-dependent survival/metabolic signaling and actin remodeling (Bichet et al., 2023). These findings should be treated as safety and pharmacology signals rather than evidence of a universal beneficial host response.

### 4.2 Inflammatory and barrier pathways

Expansion of Gram-negative pathobionts can increase lipopolysaccharide exposure. TLR4-MyD88 signaling recruits IRAK kinases and TRAF6, activates TAK1, and branches toward the IKK/NF-κB and p38/JNK/ERK MAPK pathways. The downstream program includes TNF, IL-6, chemokines, and pro-IL-1β. Parallel danger signaling can activate NLRP3, ASC-dependent caspase-1, IL-1β/IL-18 maturation, and gasdermin-D-associated pyroptosis. Sustained epithelial inflammation can increase myosin light-chain kinase activity and disrupt tight junctions. Microbial sulfonolipids can also interfere with lipopolysaccharide-TLR4 binding, illustrating why function-preserving editing may be preferable to indiscriminate depletion (Older et al., 2024).

Intracellular sensing adds a Crohn-relevant layer. Muramyl dipeptide activates NOD2-RIPK2 and downstream NF-κB/MAPK signaling. NOD1/NOD2 also recruit ATG16L1 at sites of bacterial entry, linking microbial sensing to autophagy (Cooney et al., 2010; Travassos et al., 2010). In Paneth cells, combined impairment of ATG16L1-dependent autophagy and XBP1-dependent unfolded-protein response amplifies PERK-eIF2α-ATF4 stress signaling, IRE1α-associated NF-κB activation, epithelial death, and ileitis (Adolph et al., 2013). These are mechanistic and disease-biology observations (Tier 4); phage-specific normalization has not been shown.

Evidence from non-phage microbiome interventions further supports the connection between intestinal barrier integrity and inflammatory responses. In a type 2 diabetic mouse model, Astragalus membranaceus polysaccharides were investigated in relation to glycemic outcomes and the gut microbiota–mucosal barrier axis (Chen et al., 2022). Extremophyte-derived exosome-like nanovesicles have also been studied as a multi-component approach to barrier repair, immune regulation, and microbiome modulation (Li, et al., 2026). In experimental rheumatoid arthritis, Kadsua coccinea extract was associated with TNF-alpha suppression and gut microbiota modulation (Zheng et al., 2026). These interventions differ fundamentally from bacteriophage therapy, but collectively suggest candidate epithelial and inflammatory readouts for future phage-directed studies. At the clinical-association level, diabetes was identified as a predictor of severe inflammatory bowel disease in a retrospective study integrating inflammatory, metabolic, and hepatic variables (Huang et al., 2025). Such observations justify recording metabolic comorbidity and systemic inflammation when interpreting treatment responses; they do not imply that phage treatment modifies diabetes-related IBD risk.

### 4.3 Metabolite-responsive regulatory pathways

Butyrate can promote regulatory T-cell differentiation through histone deacetylase inhibition and increased acetylation at the *FOXP3* locus (Furusawa et al., 2013). Short-chain fatty acids also signal through FFAR2/GPR43 and HCAR2/GPR109A, and epithelial butyrate oxidation supports hypoxia-inducible-factor-dependent barrier integrity (Singh et al., 2014; Kelly et al., 2015; Mann et al., 2024). Microbial tryptophan derivatives engage AhR and can increase IL-22, which activates epithelial JAK/STAT3 programs supporting antimicrobial peptides, mucus, and repair; indole derivatives can also signal through PXR (Zelante et al., 2013; Venkatesh et al., 2014). Microbial bile-acid transformations generate ligands for FXR and TGR5 and influence T-cell differentiation. 3-oxoLCA inhibits TH17 differentiation through RORγt, whereas isoalloLCA can enhance FOXP3-positive regulatory T cells (Hang et al., 2019; Song et al., 2020). TGR5 signaling can reduce macrophage inflammatory activity, and human IBD activity has been associated with loss of TGR5-activating bile-acid profiles (Won et al., 2025; Stallhofer et al., 2026; Pols et al., 2011). These pathways provide candidate downstream readouts, not established consequences of precision phage-synbiotic treatment.

Specific host-active lipid mediators provide an additional rationale for metabolite-resolved endpoints. Experimental work identified intestinal 12R-HETE as an endogenous Nur77 ligand involved in NKp46-positive group 3 innate lymphoid-cell development (Huang et al., 2026). This result provides a mechanistic example of intestinal lipid signaling but does not establish a microbial source or phage-responsive pathway. A future ecological intervention should therefore distinguish changes in microbial metabolite production from host-derived lipid signaling and verify receptor engagement rather than inferring causality from pathway enrichment alone.

## 5 Ecological replacement after selective phage perturbation

### 5.1 Colonization resistance and the successor problem

Human studies show that probiotic mucosal engraftment is person- and region-specific and cannot be inferred reliably from fecal detection alone (Tier 2) (Zmora et al., 2018). A cross-cohort analysis further found that baseline microbiome features predict *Bifidobacterium* persistence after supplementation (Goswami et al., 2026). Independent preclinical studies show that established strains can be displaced through ecological competition or vaccine-enhanced competition (Bakkeren et al., 2025; Lentsch et al., 2025). Together, these studies identify colonization resistance as a central limitation, but none demonstrates a complete phage-to-successor-to-prebiotic reconstruction sequence.

The resident phageome may also influence successor persistence. A 2025 synthesis of irritable bowel syndrome (IBS) studies described substantial interpersonal virome variability and proposed that endogenous phages could impair a beneficial strain by infecting it or facilitate its establishment by removing competitors (Rettura et al., 2025). This is an observational and mechanistic hypothesis, not evidence that a defined phageome intervention treats IBS. It nevertheless has a practical consequence: cross-host testing should evaluate both whether the therapeutic phage infects the successor and, where technically feasible, whether abundant endogenous phages from the intended recipient community can suppress that successor.

Preclinical interventions support individual components of ecological cooperation. In a chick model of multidrug-resistant *Salmonella* infection (Tier 3), phage plus *Limosilactobacillus reuteri* improved villus/crypt morphology, increased *OCLN*, *TJP1*, and *MUC2* expression, reduced IL-6, and shifted bacterial and organic-acid profiles (Choi et al., 2026). In a mouse *E. coli* K1 model (Tier 3), capsule-targeting phages selected capsule-deficient escape variants susceptible to antibody-mediated clearance and competitive exclusion by probiotic *E. coli* Nissle (Larsson et al., 2026). These studies support phage-probiotic complementarity and evolutionary steering, but they use different systems and do not validate the complete sequence proposed below.

### 5.2 Subtract-seed-feed-lock as a proposed design scaffold

Subtract-Seed-Feed-Lock (Tier 5) is used here as a development sequence, not a clinically validated protocol. Subtract denotes selective lysis, evolutionary weakening, or function editing. Seed denotes introduction or promotion of a niche-matched successor that is resistant to the therapeutic phage. Feed denotes selective nutritional support for that successor. Lock denotes persistence of the desired functional state after treatment withdrawal, potentially supported by host-microbiome feedback rather than continuing therapy.

The proposed Ecological Vacancy Window is the interval after target suppression during which adhesion sites, nutrient streams, and cross-feeding opportunities may be more accessible to a successor. Its existence, timing, and width remain unvalidated and are likely to depend on the target, phage, diet, inflammatory state, and host. The proposed Minimum Ecological Dose is the lowest phage exposure that produces a prespecified, durable functional transition rather than maximal bacterial eradication. The proposed Functional Phageability assessment asks whether a causal strain or function is selective, accessible, engineerable, ecologically replaceable, and sufficiently safe to justify development. These concepts should advance only when their components are operationally defined and prospectively tested.

### 5.3 Precision nutritional support and the compatibility gate

A successor also requires substrate access. Human prebiotic responses are baseline-microbiome dependent. In older adults with functional constipation (Tier 1), *B. animalis* subsp. *lactis* BL-99 plus fructooligosaccharides improved bowel outcomes and increased *Bifidobacterium*, with some persistence after withdrawal (Li et al., 2025); this was not a phage study. In experimental colitis, a ginger-derived exosome-like nutrient depot increased probiotic nutrient uptake under competition (Pan et al., 2025). In male mice with type 2 diabetes, an engineered *E. coli* Nissle platform linked reactive-oxygen-species tolerance to fructooligosaccharide-CaCO_3_ functionalization (Mao et al., 2026). These studies support the “Feed” concept but do not validate its integration with phage treatment.

Substrate chemistry and microbial baseline state may strongly influence the Feed stage. In vitro fermentation of blackberry polysaccharides with differing molecular weights demonstrated the importance of polymer characteristics when evaluating gut microbial fermentation responses (Li et al., 2022). A review of Chinese herbal medicine and the colorectal-cancer microbiome likewise illustrates the broad context dependence of microbiota–phytochemical interactions (Hou et al., 2025). In juvenile Macrobrachium rosenbergii, dietary fucoidan was investigated in relation to microbiota, gut health, and immune outcomes (Luo et al., 2026); the aquatic-animal model limits extrapolation to human colonic ecosystems. Together, these studies reinforce the need for compound-specific fermentation assays and model-appropriate validation; none directly demonstrates phage–successor–prebiotic compatibility.

A proposed nutrient-conditioned phagogram would test adsorption, efficiency of plating, burst size, and resistance under anaerobiosis, mucin, bile salts, selected prebiotics, probiotic-conditioned media, target-successor co-culture, and inflammatory redox conditions. The Gut Phage Biobank demonstrates that infectivity depends on environmental context (Xing et al., 2025). Accordingly, prebiotics and successors should not be appended after phage selection. They form a three-way compatibility gate: the phage must affect the causal target while sparing the successor and key commensals; the successor must compete in the post-phage niche; and the nutrient must preferentially support the successor without suppressing phage activity or feeding the pathobiont. Only combinations that pass this gate should enter *in vivo* Eco-PK/PD testing (Figure 5; Table 5).

**Figure 5:**
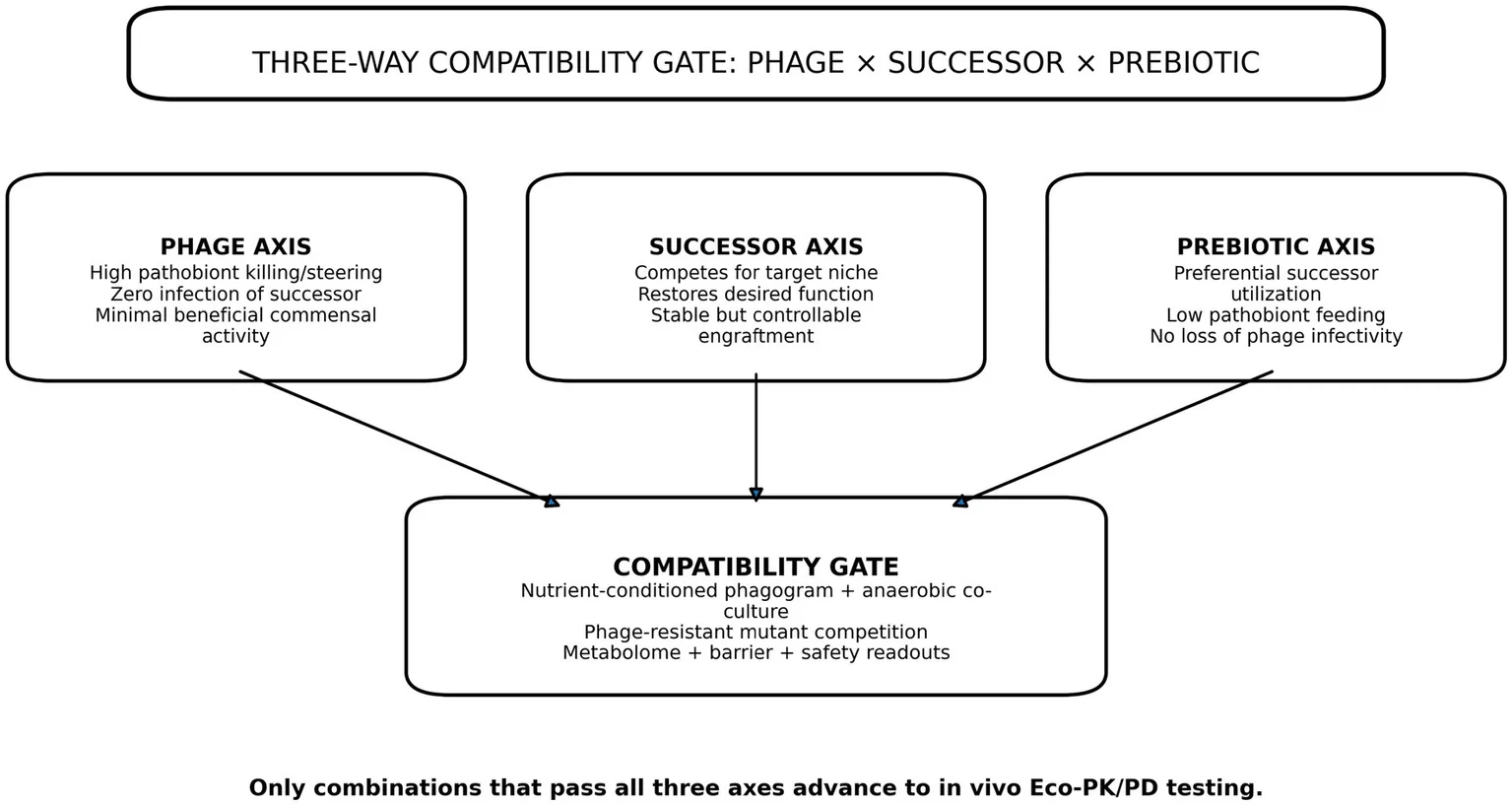
Three-way compatibility gate before *in vivo* Eco-PK/PD testing. The same terminology and advancement rule are used in Figure 1: phage, successor, and prebiotic are co-selected, and failure on any axis prevents advancement. This is an author-proposed decision framework informed by context-dependent phage infectivity, probiotic engraftment, competition, and nutrient-support studies (Xing et al., 2025; Larsson et al., 2026; Pan et al., 2025; Mao et al., 2026; Zmora et al., 2018; Bakkeren et al., 2025; Lentsch et al., 2025; Goswami et al., 2026; Li et al., 2025).

**Table 5:** Operational criteria for the proposed phage-successor-prebiotic compatibility gate.

Gate domain	Minimum advancement criterion	Required experimental context	Evidence status
Causal target	Manipulation of the strain or function changes a validated disease intermediate	Strain-resolved causal model	Established only for selected targets such as IBD-associated *K. pneumoniae* and cytolytic *E. faecalis* (Federici et al., 2022; Duan et al., 2019)
Phage-target activity	Reproducible lysis, steering, or editing under gut-relevant conditions	Anaerobiosis, mucin, bile, relevant nutrients, community context	Context dependence established; standardized gate proposed (Xing et al., 2025)
Phage-successor compatibility	Negligible infection of the selected successor	Direct cross-host testing plus receptor/genome analysis	Required design criterion; not standardized clinically
Endogenous-phage-successor compatibility	No major suppression by relevant resident phages	Patient/community virome plus isolate testing where feasible	Proposed extension motivated by IBS and engraftment literature (Zmora et al., 2018; Goswami et al., 2026; Rettura et al., 2025)
Successor competition	Successor displaces the target or its escape variant and restores a defined function	Co-culture, *ex vivo* community, then animal model	Component-level preclinical support (Larsson et al., 2026; Bakkeren et al., 2025; Lentsch et al., 2025)
Nutrient selectivity	Higher utilization by successor than by target; no material loss of phage activity	Factorial phage-target-successor-prebiotic design	Proposed; nutrient-support components shown separately (Pan et al., 2025; Mao et al., 2026; Li et al., 2025)
Functional recovery	Prespecified metabolite, barrier, or immune endpoint improves	Multi-omics plus host model	Must be demonstrated for each indication
Durability	Functional state persists after all active components are withdrawn	Longitudinal withdrawal design	Defining test of the proposed “Lock” stage

## 6 Disease-specific opportunities require evidence-matched claims

Disease areas differ sharply in causal resolution and therapeutic validation (Figure 6; Table 6). IBD and alcohol-associated liver disease contain direct preclinical examples in which a defined bacterial strain or toxin-producing phenotype was targeted with phages. Enteric infection models provide phage-probiotic proof of concept. By contrast, metabolic and neurodegenerative studies primarily identify phage-bacteria-metabolite associations or causal metabolites without testing therapeutic phages.

**Figure 6:**
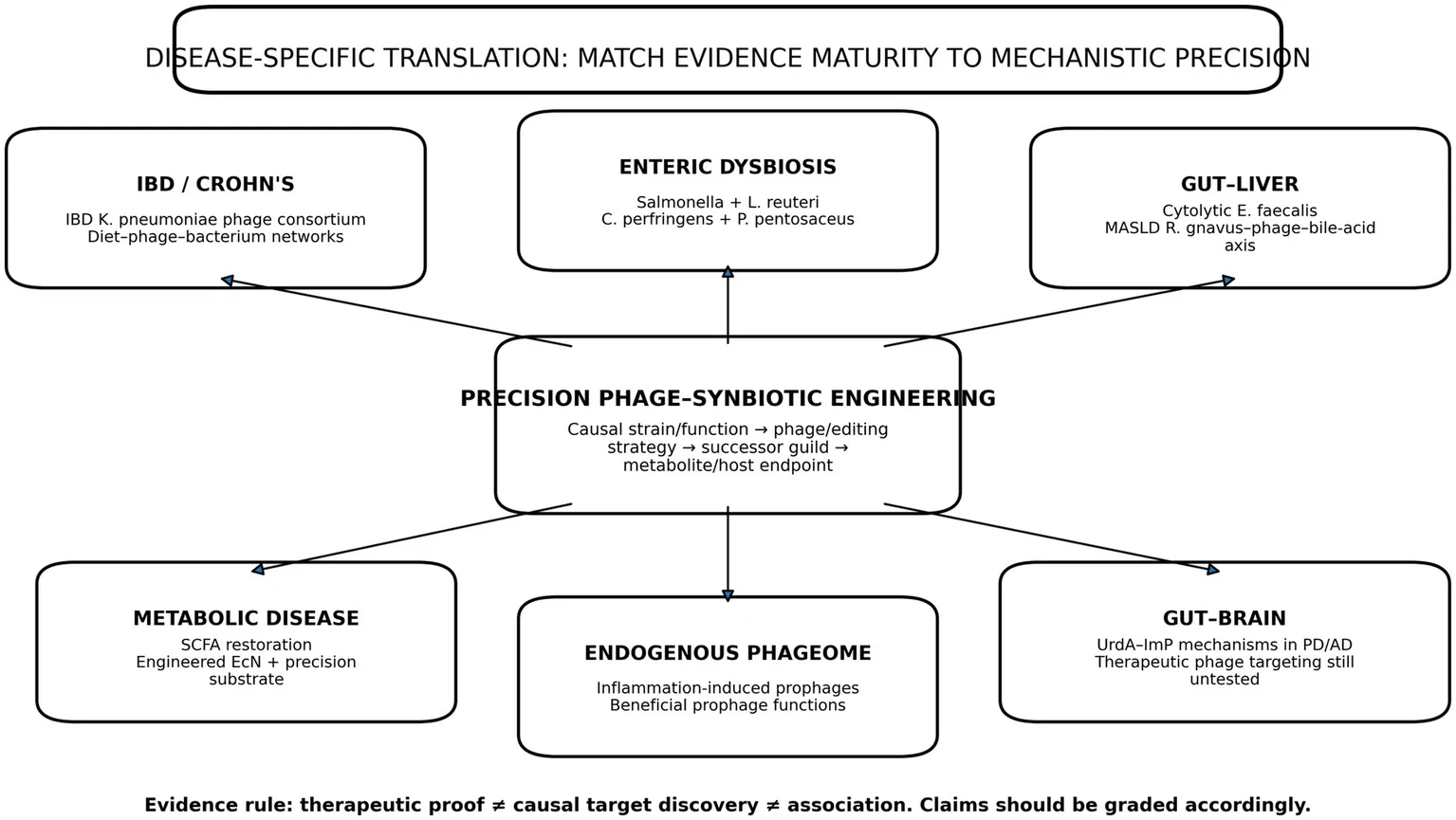
Disease-specific translational map with explicit evidence boundaries. Direct therapeutic phage evidence is concentrated in selected preclinical intestinal and gut-liver models. MASLD, IBS, and neurological applications remain at observational, mechanistic, or hypothesis-generating stages (Federici et al., 2022; Duan et al., 2019; Yang et al., 2025; Choi et al., 2026; Su et al., 2025; Zhou et al., 2026; Park et al., 2025; Vemuganti et al., 2026; Mao et al., 2024; Hegelmaier et al., 2026; Iliev et al., 2025; Rettura et al., 2025).

**Table 6:** Disease-specific phage opportunities graded by the evidence classification.

Condition	Candidate target or network	Evidence actually available	Tier	Permitted conclusion
IBD	IBD-associated *K. pneumoniae* strains	Human strain association, mouse causal validation, five-phage intervention (Federici et al., 2022)	2 + 3	Strong preclinical target-validation chain; human efficacy unknown
Crohn’s disease	Diet-AkkZT003P-*A. muciniphila*-*S. thermophilus* network	Dietary and cross-kingdom intervention model (Su et al., 2025)	3	Context-dependent ecological mechanism; not a universal target
Alcohol-associated liver disease	Cytolytic *E. faecalis*	Humanized-mouse phage intervention linked to cytolysin (Duan et al., 2019)	3	Strong preclinical toxin-directed rationale; clinical efficacy unknown
MASLD	*R. gnavus*-*F. prausnitzii*-phage-bile-acid network	Human case–control multi-omics (Zhou et al., 2026)	2	Target nomination only; therapeutic phage untested
Enteric *Salmonella* dysbiosis	*Salmonella Typhimurium*	Phage-probiotic chick model and phage-hydrogel animal model (Yang et al., 2025; Choi et al., 2026)	3	Component-level proof of concept; human intestinal pharmacology unknown
Parkinson’s disease	*urdA*-positive imidazole-propionate producers	Causal metabolite and p38γ-mTORC1 mechanism; no phage intervention (Park et al., 2025)	3 + 4	Candidate for future depletion/editing experiments only
Alzheimer-related pathology	Imidazole-propionate-producing community	Human association plus endothelial/neural experiments; no phage intervention (Vemuganti et al., 2026)	2 + 4	Metabolite-first target discovery only
IBS	Individualized virome/phage-bacteria associations	Human observational studies and synthesis (Rettura et al., 2025)	2	Stratification hypothesis; no validated phage treatment
Multiple sclerosis/amyotrophic lateral sclerosis	No validated phageable causal target	Multikingdom associations	2/5	Do not nominate therapeutic phages prematurely

### 6.1 Inflammatory bowel disease and enteric inflammation

In IBD, Federici et al. identified disease-associated *K. pneumoniae* strains, demonstrated inflammatory activity *in vivo*, and developed a five-phage consortium that reduced intestinal inflammation in mice (Tier 3) (Federici et al., 2022). This study provides an unusually complete preclinical chain from human association and strain isolation to causal testing and therapeutic intervention. It does not establish human efficacy, and future studies should include resistance, successor-community, metabolite, and withdrawal endpoints. Contemporary IBD microbiome frameworks similarly support strain- and function-resolved causality rather than broad taxonomic correction (Iliev et al., 2025).

In a Crohn’s disease model, dietary whey protein altered both the phageome and bacteriome, and AkkZT003P-mediated lysis of *Akkermansia muciniphila* was linked to an ecological context favoring *Streptococcus thermophilus* and reduced inflammation (Tier 3) (Su et al., 2025). The study illustrates diet-phage-bacterium interdependence; it does not establish that *A. muciniphila* is generally harmful or that AkkZT003P should be translated without context. In a separate *Salmonella* colitis model, compatible oral hydrogel microspheres protected and regionally released phages, producing substantial target reduction and lower inflammatory markers without antibiotic-like dysbiosis (Tier 3) (Yang et al., 2025).

### 6.2 Gut-liver and metabolic disease

In alcohol-associated liver disease, cytolytic *E. faecalis* is a functionally precise target because cytolysin directly contributes to hepatocyte injury. Phage treatment reduced intestinal cytolysin and prevented ethanol-induced liver injury in humanized mice (Tier 3) (Duan et al., 2019). The intervention acts upstream of liver injury by reducing a defined microbial toxin; clinical efficacy remains unte 

Metabolic and hematologic multi-omics studies illustrate the potential value of function-level target nomination. Lower abundance of Flavonifractor plautii and its product phytosphingosine was associated with susceptibility to metabolic disorders in a phlegm-dampness constitution study (Li et al., 2025). Integrated microbiome and metabolomic profiling also identified Cupriavidus metallidurans as a potential target in beta-thalassemia (Guo et al., 2024). These findings motivate bacterial-metabolite validation in their respective populations, but they neither establish that the implicated strains are suitable phage targets nor show therapeutic phage benefit.

For metabolic dysfunction-associated steatotic liver disease (MASLD), a 2026 case–control multi-kingdom study of 210 cases and 210 controls identified *Ruminococcus gnavus* as an enriched ecological hub, depletion of *Faecalibacterium prausnitzii* and associated phages, reduced inferred lytic control of *R. gnavus*, and altered bile-acid patterns including isodeoxycholic acid (Tier 2) (Zhou et al., 2026). These observations nominate a phage-bacterium-bile-acid network for causal testing; they do not show that an *R. gnavus* phage treats MASLD. Modified gut virome transfer has improved metabolic-syndrome-related phenotypes in obese male mice (Tier 3), but that community-level intervention cannot be equated with a defined precision phage product (Mao et al., 2024).

### 6.3 Neurological and functional gastrointestinal disorders

Parkinson’s and Alzheimer-related research currently supports causal-metabolite and host-pathway investigation, not therapeutic phage efficacy. A randomized human study of short-chain fatty acids and a prebiotic in Parkinson’s disease supports the broader tractability of gut-derived metabolic interventions, but it neither tests phages nor validates a specific phageable target (Hegelmaier et al., 2026). In multiple sclerosis and amyotrophic lateral sclerosis, multikingdom associations remain insufficient to nominate a therapeutic phage. In IBS, human virome studies identify altered and highly individualized viral communities, but causal bacteriophage targets and phage interventions remain unvalidated (Rettura et al., 2025).

The gut–brain literature provides an analogous evidence boundary. Fecal 16S rRNA profiling and multi-compartment metabolomics in APP/PS1 mice characterized interactions among microbial composition and metabolites (Cheng et al., 2022). A subsequent APP/PS1 mouse study evaluated treadmill exercise, gut microbiota remodeling, neuroinflammation, and fecal microbiota transplantation (Yuan et al., 2026). These are relevant preclinical observations for metabolite-first neurophagomic target discovery, not demonstrations that phages prevent neurodegeneration. Translation would require identification of a defined causal producer, phage susceptibility, exposure, and host-response endpoints.

## 7 Gut-brain applications: a deliberately forward-looking hypothesis

The term neurophagomic reverse translation is used here only for a proposed target-discovery workflow (Tier 5): begin with a host-active microbial metabolite, identify its biosynthetic gene and strain-level producers, and then test whether the function can be safely altered by a phage or phage-derived editor. It does not describe an established therapeutic discipline.

Imidazole propionate (ImP) illustrates this logic. In Parkinson’s disease-related work, increased *Streptococcus mutans*, the microbial urocanate reductase gene *urdA*, and plasma ImP were associated with disease. Colonization with UrdA-positive organisms increased systemic and brain ImP and produced dopaminergic pathology in experimental models. ImP activated p38γ-mTORC1 signaling, with phosphorylation of S6 and 4E-BP1; rapamycin or p38γ inhibition reduced toxicity (Tier 3/4) (Park et al., 2025). In Alzheimer-related work, higher plasma ImP was associated with preclinical cognitive and biomarker phenotypes in 1,196 cognitively unimpaired adults (Tier 2). Experimental studies then showed endothelial barrier impairment and GSK3β-associated tau hyperphosphorylation (Tier 4) (Vemuganti et al., 2026). More general animal evidence indicates that the microbiota influences blood–brain-barrier permeability and microglial maturation (Braniste et al., 2014; Erny et al., 2015).

The translational boundary is clear: no cited study has used a therapeutic phage to deplete an ImP-producing strain, edit *urdA*, lower ImP, or improve a neurological phenotype. A future experiment could compare lytic depletion with non-lethal *urdA* editing and measure ImP, microbial functional redundancy, neurovascular integrity, and pathway-specific host outcomes. Until such intervention data exist, “neurophagomics” should remain a concise hypothesis-generating subsection rather than a clinical claim.

## 8 Delivery and spatial pharmacology

Oral phage exposure is shaped by gastric acid, proteases, bile, transit time, mucus, target-density thresholds, and regional bacterial phenotypes. A sodium alginate/hyaluronic acid/Eudragit S100 microsphere system protected Salmonella phages, prolonged intestinal retention, enabled colon-responsive release, and improved outcomes in an animal colitis model (Tier 3) (Yang et al., 2025). Mucus-associated phage binding may contribute to local retention (Barr et al., 2013). These observations support formulation engineering, but regional exposure and target engagement in humans require direct measurement.

Spatial behavior is increasingly tractable in preclinical models. Phollow visualized phage transmission at single-virion resolution in the zebrafish gut (Tier 3) (Ortiz de Ora et al., 2025). Transferable Ig-like phage adhesins increased epithelial association and mouse-gut retention, with intracellular trafficking toward Golgi-associated and endoplasmic-reticulum compartments (Tier 3/4) (Apjok et al., 2026). Near-infrared reporter systems may extend preclinical imaging depth, but they are not established human gut-phage imaging tools (Hassan et al., 2026). The animal species and experimental context should accompany every extrapolation.

A future multi-compartment oral formulation could release the phage first, delay successor delivery until a measured target response occurs, and then provide sustained nutritional support. This sequence is a formulation hypothesis, not a demonstrated product (Figure 7). Its development would require component stability, absence of phage-successor antagonism, verified release kinetics, and regional Eco-PK/PD measurements.

**Figure 7:**
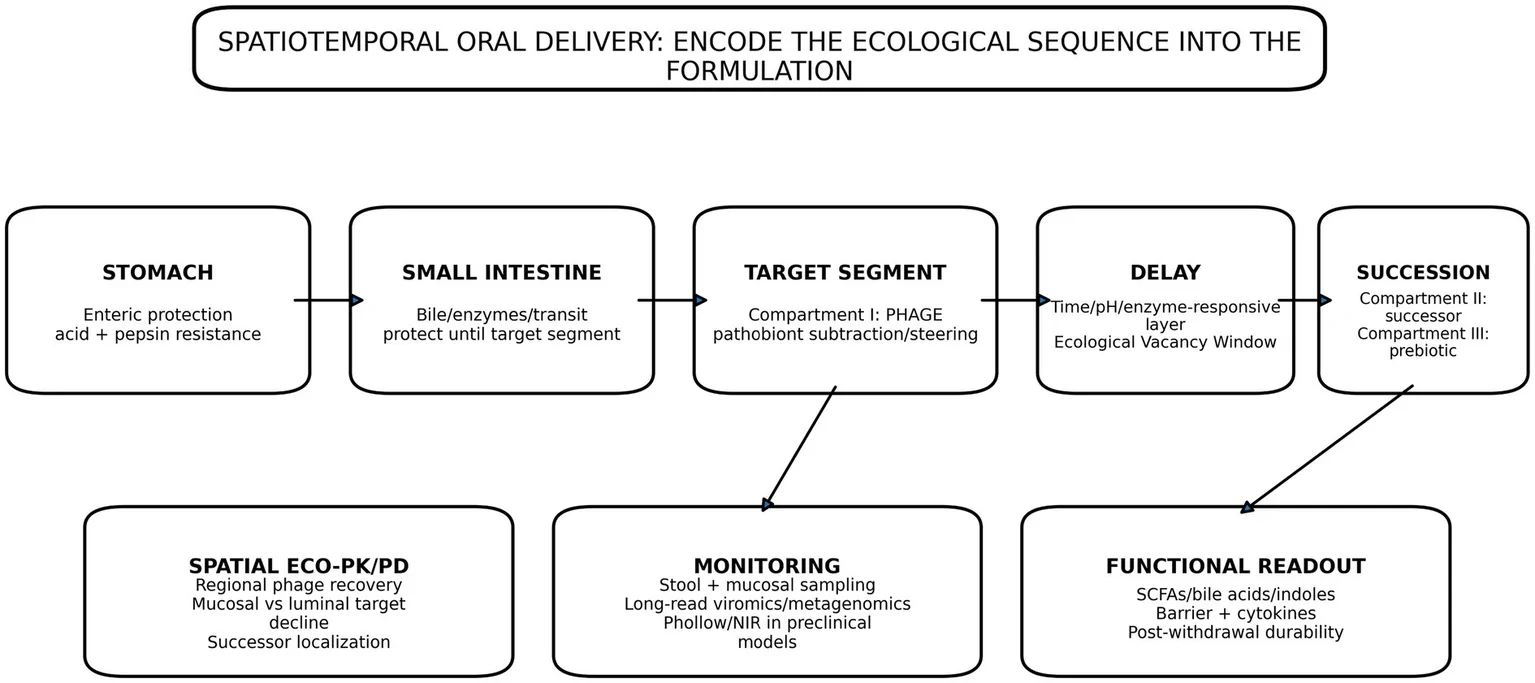
Proposed spatiotemporal delivery architecture for sequential ecological intervention. Existing animal studies support encapsulation, regional phage release, mucosal retention, and live imaging; the integrated phage-delay-successor-prebiotic dosage form remains hypothetical (Yang et al., 2025; Ortiz de Ora et al., 2025; Apjok et al., 2026; Barr et al., 2013; Hassan et al., 2026).

## 9 Eco-PK/PD and adaptive modeling are proposed measurement frameworks

Phages replicate, decay, and evolve with a living target. In dysbiosis, clinical relevance may also depend on non-target community recovery. Eco-PK/PD is therefore proposed (Tier 5) as a measurement architecture integrating phage exposure, target response, resistance, successor engraftment, community and phageome effects, metabolite recovery, host response, and post-withdrawal durability. It is not a validated pharmacometric model.

The response trajectory should extend beyond plaque-forming units versus colony-forming units and include administered dose, regional viable phage, target suppression or editing, resistance path, successor engraftment, community function, metabolite output, barrier/immune response, disease phenotype, and persistence after withdrawal. For chronic dysbiosis, maximal killing may not be optimal if it destabilizes beneficial guilds. The proposed Minimum Ecological Dose would therefore be defined prospectively as the lowest exposure meeting a prespecified functional and durability endpoint.

A microbiome-state digital twin is likewise aspirational. A future model could combine strain-resolved metagenomics, phage-host assignments, prophage state, metabolomics, diet, inflammation, candidate phages, successor traits, and nutrient utilization. It could compare intervention sequences and update predictions with longitudinal data. Existing cultured phage banks, long-read viromics, live imaging, and strain-competition studies provide component data (Xing et al., 2025; Wirbel et al., 2026; Dahlman et al., 2025; Ortiz de Ora et al., 2025; Bakkeren et al., 2025; Lentsch et al., 2025), but no clinically validated digital twin currently selects phage-synbiotic therapy. A measurable endpoint architecture is proposed without implying that its composite indices are already qualified (Table 7).

**Table 7:** Proposed Eco-PK/PD endpoint architecture.

Layer	Minimum measurement	Interpretive purpose	Current status
Phage exposure	Viable PFU, genome copies, *in situ* replication, regional recovery, mucosal retention	Distinguish administered dose from effective local exposure	Component methods available (Petersen et al., 2026; Yang et al., 2025; Ortiz de Ora et al., 2025; Apjok et al., 2026)
Target response	Absolute strain abundance and functional-gene abundance	Quantify direct pharmacodynamics	Established analytical objective; thresholds indication-specific
Resistance/steering	Whole-genome sequencing of isolates, receptor phenotype, fitness cost	Distinguish failure from useful evolutionary preconditioning	Preclinical precedent (Larsson et al., 2026)
Successor	Absolute abundance and mucosal persistence	Measure ecological replacement rather than stool transit	Human engraftment challenge established (Zmora et al., 2018; Goswami et al., 2026)
Community	Shotgun metagenomics with strain tracking and absolute quantification	Detect collateral shifts and secondary blooms	Feasible; causal interpretation remains difficult
Phageome/prophages	Lytic/temperate composition, host assignment, induction state	Detect response of the endogenous viral ecosystem	Limited by methods and database coverage (Wirbel et al., 2026; Dahlman et al., 2025; Sinha et al., 2026; Rettura et al., 2025)
Metabolome	SCFAs, bile acids, indoles, ImP, and indication-specific molecules	Link ecological change to host-active functions	Established assays; intervention-specific validation required
Barrier/immune	Occludin, claudins, tight-junction protein 1, mucin 2, calprotectin, cytokines, pathway biomarkers	Quantify host response	Mostly preclinical in phage-synbiotic settings (Yang et al., 2025; Choi et al., 2026)
Durability	Repeated measurements after all components are withdrawn	Distinguish temporary suppression from state change	Required test; integrated evidence absent
Safety	HGT, payload persistence, immune neutralization, shedding, unintended metabolites	Ecological pharmacovigilance	Development requirement (Borodovich et al., 2026; US Food and Drug Administration, National Institute of Allergy and Infectious Diseases, 2021; European Medicines Agency, 2025; Reindel and Fiore, 2017; European Medicines Agency, 2006)

## 10 Ecological pharmacovigilance, manufacturing, and regulatory feasibility

### 10.1 Ecological safety and genotoxicity

Safety assessment extends beyond confirming that a therapeutic phage genome lacks recognized virulence or antimicrobial-resistance genes. Long-read sequencing of capsid-protected DNA has detected bacterial genomic DNA consistent with generalized, specialized/lateral, and gene-transfer-agent-like mobilization in the gut virome (Tier 2) (Borodovich et al., 2026). Therapeutic products may differ from endogenous particles, but the observation supports direct measurement of residual host DNA, packaging, and transduction under relevant conditions.

Ecological pharmacovigilance is used here as a proposed longitudinal safety framework covering resistance, secondary blooms, commensal loss, prophage induction, horizontal gene transfer, payload dissemination, unexpected metabolite shifts, immune neutralization, and environmental shedding. Ecological genotoxicity is the narrower proposed category of unwanted genetic change caused by transduction, off-target editing, or mobilization of genetic elements. Therapeutic perturbation itself should be tested for changes in prophage induction, bacterial competition, and gene transfer (Wirbel et al., 2026; Dahlman et al., 2025; Sinha et al., 2026; Gao et al., 2025; Borodovich et al., 2026).

### 10.2 Good manufacturing practice and product control

Manufacturing must control the biological variability that gives phages therapeutic value. A development program should establish qualified bacterial master and working cell banks; sequence and characterize each phage and production host; demonstrate identity, potency, purity, sterility, and absence of adventitious agents; limit endotoxin, exotoxin, residual host-cell DNA/protein, and process contaminants; and validate purification, formulation, storage, shipping, and administration conditions. Batch-to-batch consistency is particularly challenging for cocktails because the potency and impurity profile of each component and the combined product must remain controlled. Stability studies should measure viable titer, aggregation, adsorption competence, genome integrity, and activity throughout shelf life. Synbiotic co-formulations add interaction risks among phages, live bacteria, nutrients, excipients, and staged-release compartments.

Resistance, patient-to-patient pharmacokinetic variability, anti-phage antibodies, and environmental shedding require prospective plans rather than *post hoc* observation. Dynamically updated cocktails create an additional comparability problem: replacing one phage may be clinically rational when bacterial susceptibility changes, but identity, manufacturing, safety, and potency data must support the updated component and final mixture. These manufacturing and surveillance requirements are integrated in a single translational matrix (Table 8).

**Table 8:** Integrated manufacturing, regulatory, and ecological pharmacovigilance requirements.

Domain	Principal failure mode	Minimum control strategy	Translational implication
Phage and host identity	Misidentified phage, lysogeny/toxin/ARG risk, unstable host	Whole-genome sequencing, curated annotation, qualified phage and bacterial banks	Required for traceability and reproducibility (US Food and Drug Administration, National Institute of Allergy and Infectious Diseases, 2021; European Medicines Agency, 2025; Reindel and Fiore, 2017)
Purity and contamination	Endotoxin, exotoxin, host-cell DNA/protein, adventitious agents, sterility failure	Validated purification and release specifications	Major CMC and safety determinant (US Food and Drug Administration, National Institute of Allergy and Infectious Diseases, 2021; Reindel and Fiore, 2017)
Potency	Loss of viable titer or adsorption competence	Qualified plaque/kill assays plus component-specific and cocktail potency	Must remain linked to intended target and release conditions (US Food and Drug Administration, National Institute of Allergy and Infectious Diseases, 2021; European Medicines Agency, 2025)
Stability	Titer loss, aggregation, genome damage, formulation interaction	Real-time/accelerated stability, shipping and in-use studies	Especially complex for live synbiotic and staged-release products (European Medicines Agency, 2025)
Batch consistency	Variable component ratio or impurity profile	Validated process controls and lot comparability	Critical for multi-phage cocktails (US Food and Drug Administration, National Institute of Allergy and Infectious Diseases, 2021; European Medicines Agency, 2025)
Resistance and PK variability	Non-response, rapid rebound, variable replication	Baseline susceptibility, longitudinal isolates, regional PK, adaptive plan	Adaptation must not bypass comparability requirements
Immune response	Neutralization or inflammatory reaction	Anti-phage antibodies, cytokines, complement, dose/route assessment	May alter repeat dosing and patient selection
Successor compatibility	Therapeutic or endogenous phage suppresses successor	Therapeutic cross-host testing and feasible endogenous-phage challenge panel	Required for any ecological replacement claim (Zmora et al., 2018; Goswami et al., 2026; Rettura et al., 2025)
Genetic/ecological spread	Transduction, payload transfer, prophage activation, shedding	Capsid-DNA sequencing, off-target assays, long-read viromics, environmental monitoring	Supports ecological pharmacovigilance (Wirbel et al., 2026; Dahlman et al., 2025; Sinha et al., 2026; Gao et al., 2025; Borodovich et al., 2026; European Medicines Agency, 2006)
Cocktail update	Replacement component changes identity, potency, or risk	Prespecified comparability protocol and supporting CMC data	Central hurdle for dynamically updated products (US Food and Drug Administration, National Institute of Allergy and Infectious Diseases, 2021; European Medicines Agency, 2025)

### 10.3 FDA and EMA considerations

In the United States, FDA presentations describe therapeutic phages for infectious-disease indications as investigational biological products evaluated under an Investigational New Drug application. Chemistry, manufacturing, and controls information should support product identity, testing, lot release, safety, purity, potency, and consistent manufacture. FDA workshop materials identify inadequate endotoxin, exotoxin, sterility, genomic, and lot-release information as recurrent early-development deficiencies and state that adding or replacing cocktail components requires supporting chemistry, manufacturing, and controls information (US Food and Drug Administration, National Institute of Allergy and Infectious Diseases, 2021). FDA authors have also emphasized transduction, resistance, and endotoxin release as product-development concerns (Reindel and Fiore, 2017). These materials do not create a separate streamlined pathway for complex gut-ecosystem products.

In the European Union, the European Medicines Agency’s 2025 draft guideline on quality aspects of human phage therapy medicinal products addresses manufacture, starting materials, characterization, specifications, analytical control, reference standards, finished-product control, and stability, including characterization of genetically or chemically modified phages (European Medicines Agency, 2025). Products containing or consisting of genetically modified organisms may also require an environmental risk assessment under applicable European guidance (European Medicines Agency, 2006). The exact classification and dossier requirements depend on the construct, indication, mode of action, and jurisdiction; sponsors should obtain product-specific scientific advice.

Multi-phage and phage-synbiotic products are therefore more complex than a traditional single active substance. Developers must define whether components are co-formulated or co-administered, which component drives the claimed effect, how interactions are controlled, how each component is released and tested, and how a post-approval phage substitution would be managed. Early engagement with regulators is essential because established FDA and EMA principles apply, but a validated approval pathway for dynamically personalized phage-successor-prebiotic reconstruction has not been demonstrated.

## 11 Methodological limitations of gut virome research

Personalized Eco-PK/PD and adaptive models are constrained by how incompletely the gut virome is measured. First, viral reference databases remain incomplete, leaving a substantial fraction of sequences unclassified and limiting functional annotation. Second, linking a phage contig to its bacterial host is uncertain when culture, CRISPR spacer matches, proximity ligation, long reads, or co-abundance evidence disagree. Third, bulk metagenomics and virus-like-particle-enriched sequencing capture different fractions: bulk sequencing retains integrated prophages and cell-associated viral DNA but can be dominated by bacterial DNA, whereas virus-like-particle workflows enrich extracellular particles but introduce filtration, nuclease, extraction, and amplification biases.

Fourth, interpersonal and temporal variability is substantial, as shown in longitudinal phageome studies and IBS syntheses (Wirbel et al., 2026; Rettura et al., 2025). A universal reference phageome may therefore be less informative than within-person baselines. Fifth, fecal sampling incompletely represents mucosal and regional viral ecology. Sixth, relative abundance can create apparent changes when total microbial load shifts; absolute quantification and internal standards should accompany compositional data. Seventh, bioinformatic predictions of lytic versus temperate state, auxiliary genes, or host range require experimental validation. Finally, association networks cannot distinguish whether phages drive bacterial change, respond to bacterial abundance, or covary with diet, medication, inflammation, and transit.

These limitations do not preclude personalized therapy, but they define minimum methodological safeguards: pre-registered sampling and processing, extraction controls, paired bulk and virus-like-particle workflows when feasible, strain-resolved bacterial data, explicit host-assignment confidence, absolute quantification, longitudinal within-person sampling, and culture- or community-based validation of key phage-host links. Digital-twin predictions should report uncertainty propagated from each data layer rather than returning a single deterministic treatment sequence.

## 12 Experimentally testable priorities

The proposed framework will be useful only if it generates discriminating experiments. Priorities should test whether phage-first treatment creates a measurable opportunity for successor establishment, whether a prebiotic alters phage or target behavior, whether resistance produces a therapeutically favorable phenotype, and whether benefits persist after intervention withdrawal (Table 9).

**Table 9:** Priority experiments and falsifiable outcomes.

Experiment	Primary hypothesis	Minimum design/readouts	Result that would challenge the hypothesis
Sequential timing study	A post-phage Ecological Vacancy Window improves successor establishment	Phage-before, simultaneous, and multiple-delay arms; absolute target and mucosal successor abundance	No timing-dependent difference or increased undesirable recolonization
Full factorial Subtract-Seed-Feed study	Components interact to improve function beyond single and paired treatments	All component combinations; barrier, immune, metabolite, resistance, and withdrawal endpoints	No interaction, worse safety, or benefit explained by one component alone
Nutrient-conditioned phagogram	Diet/prebiotic changes phage susceptibility or resistance	Adsorption, EOP, latent period, burst size, receptor expression under gut-relevant conditions	Standard-broth results fully predict community performance
Resistance-steering competition	Phage-selected escape permits successor displacement	Escape-genotype mapping, fitness, virulence, antibody sensitivity, co-culture competition	Escape retains virulence/fitness or blocks successor establishment
Endogenous-phage-successor challenge	Recipient phageome affects successor persistence	Successor exposure to representative recipient viromes plus community validation	Endogenous-phage profile does not predict establishment
*urdA* depletion versus editing	Lowering ImP improves defined host pathways while editing preserves more ecological function	ImP, producer redundancy, p38γ-mTORC1, barrier/neural readouts	ImP unchanged, compensatory production, or no pathway benefit
*R. gnavus* network perturbation in MASLD	Restoring lytic control changes bile acids and liver phenotype	Defined phage, absolute bacterial/phage counts, bile acids, liver endpoints	Target reduction without metabolic or hepatic improvement
Prophage-state intervention	Modifying induction interrupts inflammation-phageome feedback	Long-read viromics, induction assays, cytokines, histology, HGT	Increased inflammation, harmful mobilization, or no causal effect
GMP-representative staged formulation	Release sequence remains stable and compatible at scale	Release kinetics, component potency/purity, stability, regional recovery	Loss of titer, successor killing, nutrient interference, or batch variability
Withdrawal/state-persistence study	A reconstructed functional state persists safely without dosing	Longitudinal absolute multi-omics, host outcomes, shedding, HGT	Rapid rebound, secondary dysbiosis, or persistent safety signal

For neurological applications, the appropriate first step is not a clinical “neurophage” claim. A controlled preclinical comparison of lytic depletion and non-lethal *urdA* editing should quantify ImP, functional redundancy among producers, p38γ-mTORC1 signaling, neurovascular integrity, and neuronal outcomes (Park et al., 2025; Vemuganti et al., 2026). For MASLD, the *R. gnavus*-phage-bile-acid network requires causal perturbation before therapeutic nomination (Zhou et al., 2026). For colitis, selective prophage induction versus stabilization could test whether an inflammation-prophage feedback loop is pathogenic or adaptive (Dahlman et al., 2025; Sinha et al., 2026; Gao et al., 2025).

Withdrawal should be built into every reconstruction experiment. A benefit that disappears immediately when dosing stops demonstrates pharmacological suppression, not durable ecological state control. Conversely, persistence should not automatically be considered beneficial; it must be accompanied by absence of secondary blooms, harmful gene transfer, adverse metabolites, and environmental spread.

## 13 Conclusion

The current evidence supports a focused conclusion. Selective phage targeting can modify defined bacterial populations in experimental systems, and early human studies support oral feasibility, gastrointestinal recovery, short-term safety, and microbiome-sparing pharmacology for specific products. Preclinical studies also show that phages can target disease-associated strains or functions, steer escape phenotypes, deliver genetic or protein payloads, cooperate with probiotics, and be protected by regional oral formulations. These achievements justify continued development of precision phage therapeutics.

What has not been demonstrated is equally important. No integrated human study has shown that phage subtraction followed by successor engraftment, precision nutritional support, and withdrawal produces a deliberately programmed, durable ecological state. Functional Phageability, Minimum Ecological Dose, Ecological Vacancy Window, nutrient-conditioned phagograms, Eco-PK/PD, ecological pharmacovigilance, neurophagomic reverse translation, and microbiome-state digital twins remain proposed operational frameworks. Their value will depend on prospective definitions, falsifiable thresholds, reproducibility across laboratories, and regulatory qualification where relevant.

The near-term translational objective should therefore be evidence-matched precision: select targets with causal support, characterize the endogenous phageome and likely resistance paths, manufacture consistent and well-controlled products, measure regional exposure and absolute target response, and monitor ecological and genetic safety. The longer-term objective, durable ecological reconstruction—should be tested rather than presumed. Subtract-Seed-Feed-Lock provides one structured way to ask that question, but the decisive endpoint is not disappearance of a bacterium; it is a reproducible and safe improvement in microbial function and host physiology that persists after treatment ends.
